# The development of the own-race advantage in school-age children: A morphing face paradigm

**DOI:** 10.1371/journal.pone.0195020

**Published:** 2018-04-10

**Authors:** Sarina Hui-Lin Chien, Chu-Lik Tai, Shu-Fei Yang

**Affiliations:** 1 Graduate Institute of Neural & Cognitive Sciences, China Medical University, Taichung, Taiwan; 2 Graduate Institute of Biomedical Sciences, China Medical University, Taichung, Taiwan; Bournemouth University, UNITED KINGDOM

## Abstract

Previous studies examining the other-race effect in school-age children mostly focused on recognition memory performance. Here we investigated perceptual discriminability for Asian-like versus Caucasian-like morph faces in school-age Taiwanese children and adults. One-hundred-and-two 5- to 12-year-old children and twenty-three adults performed a sequential same/different face matching task, where they viewed an Asian- or a Caucasian-parent face followed by either the same parent face or a different morphed face (containing 15%, 30%, 45%, or 60% contribution from the other parent face) and judged if the two faces looked the same. We computed the *d’* as the sensitivity index for each age groups. We also analyzed the group mean rejection rates as a function of the morph level and fitted with a cumulative normal distribution function. Results showed that the adults and the oldest 11-12-year-old children exhibited a greater sensitivity (*d*’) and a smaller discrimination threshold (*μ*) in the Asian-parent condition than those in the Caucasian-parent condition, indicating the presence of an own-race advantage. On the contrary, 5- to 10-year-old children showed an equal sensitivity and similar discrimination thresholds for both conditions, indicating an absence of the own-race advantage. Moreover, a gradual development in enhancing the discriminability for the Asian-parent condition was observed from age 5 to 12; however, the progression in the Caucasian-parent condition was less apparent. In sum, our findings suggest that expertise in face processing may take the entire childhood to develop, and supports the *perceptual learning view* of the other-race effect—the own-race advantage seen in adulthood likely reflects a result of prolonged learning specific to faces most commonly seen in one’s visual environment such as own-race faces.

## Introduction

The human face carries abundant visual information and social cues. In daily encounters, we automatically attend to people’s gender, age, race, and facial expression, and these characteristics may well influence our social evaluations. For example, adults belonging to one racial group typically find it difficult to recognize or memorize faces of other racial or ethnic groups than those of their own [1]. This phenomenon refers to as the “other-race effect” (ORE), or interchangeably the “own-race advantage” (ORA), reflecting the relative ineptness at processing individual faces of unfamiliar races or ethnic groups [2]. To date, the other-race effect has been reliably reported across ethnic groups (e.g., [3–7]) and the effect is robust under a variety of experimental conditions, including standard recognition memory tasks [8], naturalistic eyewitness memory paradigms [9,10], and a perceptual encoding-based face discrimination task [7,11,12].

In literature, the ORE or ORA has been demonstrated mostly as a bias in recognition memory where people can better retrieve own-race faces than other-race faces over certain retention intervals; this is particularly true for the developmental ORE literature in children. However, the existence of a recognition memory bias does not exclude the possibility that an own-race advantage may exist in the earlier perceptual encoding stage. In line with the broadly-defined perceptual expertise or learning view of the ORE (for a review see Meissner & Brigham [2]), individuals may see own-race faces as perceptually more distinctive than those faces with less experience. Using a sequential face matching task requiring a minimal load on memory retention, Walker and Tanaka demonstrated an own-race encoding advantage for Asian adults living in Canada and Canadian Caucasian participants [7]. The stimuli were a continuum of face images created by morphing an East Asian parent face with a Caucasian parent face. Their results showed that Asian participants performed better in the Asian-parent condition, whereas Caucasian participants were better at the Caucasian-parent condition. Using a similar morphing face matching task, Chen et al. examined the perceptual discriminability for Asian-parent and Caucasian-parent conditions in Taiwanese adults and found a smaller threshold for the Asian-parent condition than that for the Caucasian-parent condition, indicating cross-cultural evidence for the own-race encoding advantage [12]. Studies based on eye movement evidence also supported the own-race encoding bias. Goldinger et al. reported that while encoding other-race faces, both their Caucasian and Asian participants made fewer (and longer) fixations, preferentially attended to different sets of features, and their pupils were more dilated, as compared to the own-race faces [13]. These results suggested the presence of own-race advantage (or bias) during initial perceptual encoding; relative to own-race face encoding, other-race encoding requires greater effort, which may reduce vigilance in some participants.

### The other-race effect in infancy

When does such a bias for own-race faces emerge in life? In the last decade, convergent evidence from cross-cultural studies has pointed to an early onset of the ORE in the first year of life. Studies based on preferential looking methods revealed that 3-mo-old Caucasian infants (but not newborns) exhibited a spontaneous looking preference for the Caucasian face when paired with other-race faces [14]. Likewise, 3-mo-old Chinese infants showed spontaneous looking preferences for Chinese faces [15]. Studies based on the familiarization/novelty preference procedure and with cropped faces (i.e., without external facial cues) revealed that infants at 3 or 4 months are readily better at differentiating among own-race faces than other-race faces [16–20], and the early own-race discrimination advantage maybe gender-dependent that at 3–4 months, infants showed an ORA for female faces only [21]. On the other hand, studies based on habituation paradigm and with non-cropped full faces showed that the other-race effect is absent at 3 months and seems to emerge between 3 and 9 months [22,23]; by 9 months of age, infants become unable to discriminate faces from other races (see a different interpretation by Markant et al [24]). Moreover, eye movement data also showed that 3-month-old biracial and monoracial infants scanned differently [25] and 6- to 9-month-old infants exhibited differential fixation patterns and scan paths for own- and other-race faces [26]. This indicates a difference in perceptual encoding for familiar vs. unfamiliar race faces. Last but not least, the observed other-race effect in infancy seems to be highly plastic and rapid-learning; infant’s ORE can be eliminated after minutes of exposure to a few unfamiliar other-race faces [16, 27].

### Development of the other-race effect in childhood

Since the ORE has an early inception in the first year, one may wonder whether a continuous developmental trajectory of the ORE exists from infancy to childhood. Although the empirical research with infants and toddlers aged between 1 and 3 is lacking, several studies explored the other-race effect in preschoolers, children, and teenagers. Up to date, two studies reported the presence of the other-race effect in 3 year-olds. Using a simultaneous matching task, Sangrigoli et al. tested 3-, 4- and 5-year-old Caucasian children’s face recognition by asking them either pointing to the target face (i.e., the 3-year-olds) or making a key press (i.e., 4- and 5-year-olds) [16]. Results showed that 4- and 5-year-olds exhibited an ORE, but the 3-year-olds’ performance was at floor level. After extending the target presentation time to1000ms, the 3-year-olds were then able to show an ORE. With a forced-choice-paradigm, Suhrke et al. asked 3-year-old German and Cameroon children to recognize female Caucasian or African faces (with visible external contours, hairs, and ears)[28]. They found that German children’s overall performance was better than Cameroonians’, but both groups exhibited the other-race effect—they were better at recognizing individual faces from their own ethnic groups.

The onset of a clear-cut ORE in childhood remains inconclusive. Pezdek et al. tested 5-, 8-year-olds and adults of African American or European American descent with a 6-person line-up recognition memory task one day later [29]. They found that children in both age groups showed better own-race identification than other-race identification, indicating the presence of ORE from 5 years on. Using an old/new face recognition paradigm, De Heering et al. also reported an ORE for Caucasian faces as compared to Asian faces in Belgium Caucasian children from 6 years on [30]. However, an earlier study based on a standard recognition task by Goodman et al. failed to uncover the cross-racial effects in preschoolers [31].

Consistent with Goodman et al.[31], two studies also reported an absence of ORE in preschoolers, but a presence in older children aged beyond 8 or 9 years. Chance et al. tested European American 6- to 20-year-olds with White and Japanese faces using a standard old/new recognition task and revealed three critical findings [32]. First, the youngest group (7- and 8-year-olds) did not exhibit the ORE; only the older children (11- and 12-year-olds) and adults showed a clear ORE. Second, the recognition accuracies for both White and Japanese faces generally improved with age, but the progress for the own-race faces was more prominent. Lastly, the magnitude of ORE reaches its peak in adulthood. In a similar vein, Goodman et al. reported a multi-nation study showing that the ORE was absent in younger children [33]. They tested Caucasian children and adults of European ancestry, and biracial (Caucasian–African American) children and adults living in the United States with a standard memory recognition task of Asian, African, and Caucasian faces. Regardless of ethnic or biracial background, 8- to 10-year-olds, 12- to 14-year-olds, and adults recognized own-race faces better than other-race faces; whereas 5- to 7-year-olds recognized all face types equally well. Recently, Ding et al. used the functional Near-infrared Spectroscopy (fNIRS) to investigate the neural correlates of 7- to 13-year-old children's face processing and their behavioral performance on own- versus other-race recognition memory [34]. Although they did not find significant behavioral differences in the recognition accuracy for own- versus other-race faces in this age range, the fNIRS data revealed that with increased age, the [oxy-Hb] activity differences between own- and other-race faces underwent significant changes in two cortical areas (BA9 and BA18).

### The present study

To sum up, previous studies on the development of ORE in children mostly relied on the standard old/new recognition memory task. While the own-race advantage is stable in adulthood, the inconsistent results in children suggested that the own-race advantage may be highly plastic in childhood and particularly malleable in infancy and preschool age. Methodologically, the contradictory findings could reflect various procedural discrepancies among studies, such as the length of memory retention intervals, or the encoding time in the learning phase. Another major confounding factor could be the *non-equal physical similarities* between the selected faces from two different races. For example, it is not impossible that, in one study, young Caucasian children showed better recognition for Caucasian faces might be due to the selected Caucasian faces were more dissimilar than the Asian ones. But in another study where the physical similarity between the chosen race faces was equated, children in that study did not exhibit a reliable ORE at the same developmental age.

In light of this, the present study employed the morphing face paradigm to examine the encoding advantage hypothesis in Taiwanese school-aged children and adults as a comparison. We aimed to characterize the development of the other-race effect in children aged between 5 and 12 with a sequential same/different face matching task, which put more weight on perceptual discriminability than memory retention or retrieval capacity. We adopted the morphing paradigm with cropped female faces for several methodological considerations. First, the morphing technique allows for better control over the physical similarity between two selected faces. Secondly, we eliminated the external facial cues to encourage the participants to focus on the internal features and the configural information within the face. Lastly, the morphing technique created a linear continuum of very subtle changes suitable for estimating perceptual discriminability at near threshold level. In other words, we can calculate the minimum physical difference needed to produce a detectable perceptual change (e.g., JND) with curve fitting procedures, and hence to chart the developmental progression of the perceptual discriminability for own- and other-race faces in children aged 5 to 12 and young adults.

## Methods

### Ethics statement

The adult participants and the parents of the child participants were informed of the general purpose and the experimental procedures of the study. Written consent (or parental consent) of the participants were obtained before the experiment. The protocol of the present study was approved under the Clinical Trial/Human Research Approval by the Research Ethics Committee, China Medical University and Hospital, Taichung, Taiwan. (The protocol number: MOST 103-2410-H-039-002-MY3, and the CMUH REC number: CMUH103-REC3-055).

### Experiment 1: Adult’s study

#### Participants

A total of 23 adults (12 males, mean age = 21.62 years) participated the experiment. The majority of the subjects were undergraduate and graduate students recruited from China Medical University via campus-wide advertisement. Informed written consent was obtained before the experiment. All participants were native Chinese speakers and had a normal or corrected-to-normal vision (20/20). None of the participants had the experience of living in countries where Caucasian is the majority race more than six months. The observers were naïve to the purpose of the experiment and were tested individually in a quiet, dimly lit room. Each participant received both the Asian- and Caucasian-parent conditions, and the test order was counterbalanced among participants. Each participant received cash compensation at the end of the experiment.

#### Apparatus and stimuli

A desktop computer (Acer Veriton M460) with 22” LCD monitor (Chimei CMV 221) and E-Prime Professional 2.0 software (Psychological Software Tools, Sharpsburg, PA) were used to run the experiment. We adopted three Asian and three Caucasian female faces as the stimuli. The Asian faces were selected from the Taiwanese Facial Expression Image Database, TFEID [35], while the Caucasian faces were from the NimStim Face Stimulus Set [36]. The selected face images were with a frontal pose, neutral expression, and with no glasses, or hair covering the forehead. All faces were converted to gray-scale images and oval-cropped to remove the external cues such as the ears, the hair, and clothing information by Windows 7 Paint and PhotoImpact 10 (Ulead System, Taipei).

**Fig 1:**
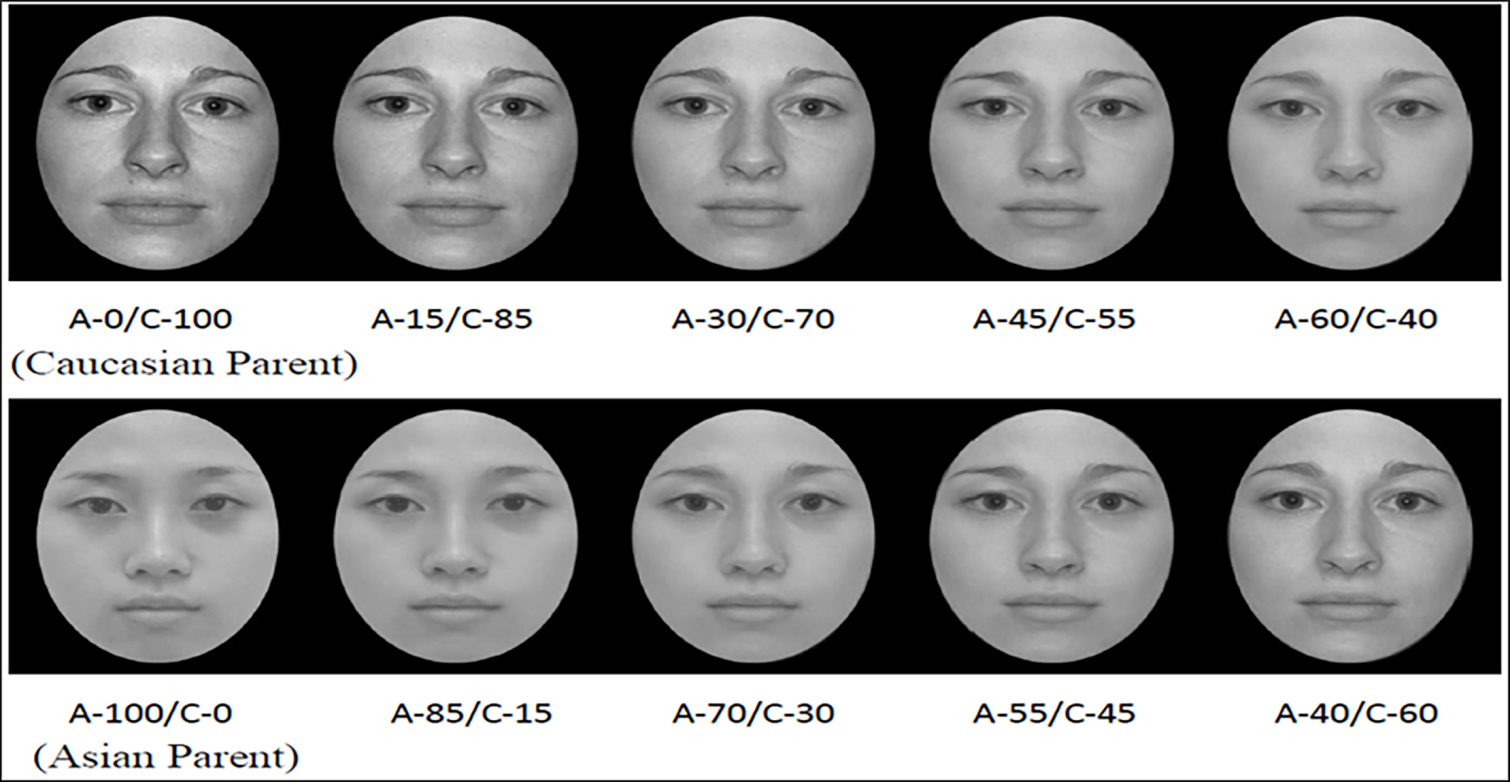
Morphed female faces stimuli. An example of a set of linearly morphed female faces produced by image averaging of one Asian- and Caucasian-parent face pair. The numerator indicates the percent contribution to the morphed face from Asian parent and the denominator indicates the percent contribution from the Caucasian parent.

The six female faces formed three pairs (each Asian female face paired with a Caucasian female face), we then used FantaMorph 5 Deluxe (Abrosoft Co.) to create three sets of morphed images on a linear continuum between the paired faces. A total of 65 key points for the facial features were kept constant for each of the oval-cropped Asian- and Caucasian-parent faces, which included 11 points on the mouth, 6 points on each eye, 10 points on the nose, 4 points on each eyebrow, and 24 points for the contour of the face. The FantaMorph program generated control points for a series of morph faces by moving 15%, 30%, 45%, and 60% of the total distance along the vector that connected corresponding control points in the Asian-parent and Caucasian-parent faces. As shown in Fig 1, the program produced a gradient morph faces, which were A100/C0 (Asian-parent face), A85/C15, A70/ C30, A55/C45, A40/C60 for the Asian-parent condition, and A0/ C100 (Caucasian-parent face), A15/C85, A30/C70, A45/C55, and A60/C40 for the Caucasian-parent condition. Note that the numerator indicated the percent contribution to the morphed face from the Asian-parent, while the denominator indicated the percent contribution from the Caucasian-parent.

#### Design and procedure

**Fig 2:**
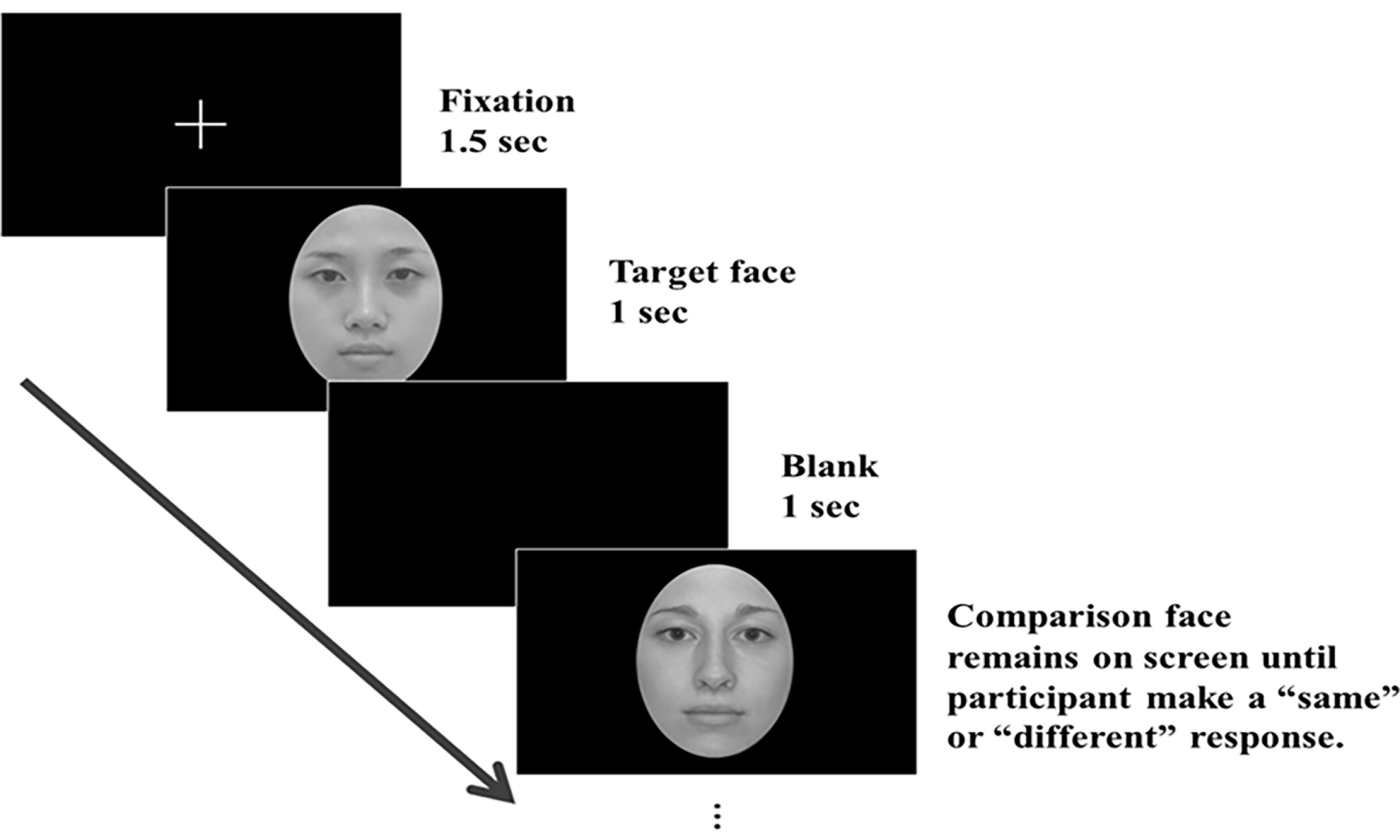
Illustration of a sample trial of the sequential same/different face matching task.

The Adult Experiment incorporated two independent within-subject factors: the race condition (Asian-parent vs. Caucasian-parent) and the morph level of the face stimuli (0%, 15%, 30%, 45%, 60%). The experiment contained two race blocks, and the test order was counterbalanced among participants. Each block included 72 trials presented in random order (a total of 144 trials). Fig 2 illustrates a sample trial of the same/different face matching task. Each trial began with a fixation cross for 1.5 seconds, followed by a target face (which was always the Asian-parent face or the Caucasian-parent face) for one second. After a one-second blank, a comparison face appeared which was either the same parent face or a different morphed face with equal stimulus probability. The participant was asked to judge whether the comparison face looked like the same person’s or a different person’s face from the target face. The comparison face remained on the screen until the participant made a keypress response and the next trial began. The participant pressed “1” (labeled as “same”) if s/he perceived the two face images as being the same, while pressed “3” (labeled as “different”) if s/he perceived the two faces as being different. Among the 72 trials in a race block, 36 were the “physically same” trials; the comparison faces were exactly the same Asian- (A100/C0) or Caucasian-parent faces (A0/ C100). The other half was the “physically different” trials. The comparison faces were four different morphed faces each appeared 9 times, A85/C15 (15%), A70/C30 (30%), A55/C45 (45%), and A40/C60 (60%) for the Asian-parent condition, and A15/C85 (15%), A30/C70 (30%), A45/C55 (45%), and A60/C40 (60%) for the Caucasian-parent condition. The dependent variable was participant’s “rejection rate,” defined as the percentage of judging the comparison face as different from the target face.

### Experiment 2: Children’s study

#### Participants

A total of 107 participants (48 boys, 59 girls), aged between 4.9 and 13.4 years, participated the study. The majority of the children were recruited from the Taichung Metropolitan Area via a parenting club on Facebook and Internet advertisements. Informed parental consent was obtained before the experiment. All child participants were native Chinese speakers and had a normal or corrected-to-normal vision (20/20). None of the child participants had the experience of living in countries where Caucasian is the majority race by their parent’s report. The child participants were tested individually in a quiet, dimly lit room. Each participant received both the Asian-parent and Caucasian-parent conditions, and the test order was counterbalanced among participants.

The participants were divided into four age groups: 5- and 6-year-olds, 7- and 8-year-olds, 9- and 10-year-olds, and 11- and 12-year-olds. In the youngest 5- and 6-year-old group, 28 children were tested, two of them were excluded from the final data due to procedural errors (1) and a response bias (1) (i.e., who only pressed the “different” key throughout the experiment). The final sample size was 26 (12 boys, 14 girls), with a mean age of 6.17 years (range between 4.99 and 6.97 years old). In the 7- and 8-year-old group, 30 children were tested, but three of them were excluded from the final data due to procedural errors (1) and a high false alarm rate (2) (i.e., greater than 50% of the physically same trials (0% morph level) being responded as “different”). The final sample size was 27 (13 boys, 14 girls) with a mean age of 7.96 years (range between 7.00 and 8.99 years). In the 9- and 10-year-old group, 28 children (12 boys, 16 girls) completed the study, and all of them retained in the final data set. Their mean age was 9.83 years (range between 9.00 and 10.86 years). In the eldest 11- and 12-year-old group, 21 children (10 boys, 11 girls) were tested and retained in the final data set. Their mean age was 11.55 years (range between 11.00 and 13.04 years old).

#### Apparatus and stimuli

The apparatus and the face stimuli were the same as in the Adults’ Study.

#### Design and procedure

The Children’s Study adopted a mixed design. The between-subject factor was age groups (5- and 6-year-olds, 7- and 8-year-olds, 9- and 10-year-olds, and 11- and 12-year-olds), while the within-subject factors were race (Asian-parent vs. Caucasian-parent) and the morph level of the face stimuli (0%, 15%, 30%, 45%, and 60%). The procedure of the sequential face matching task was the same as in the adult study. Likewise, the dependent variable was the “rejection rate,” defined as the percentage of judging the comparison face as “different”.

## Results

### Overview of data analyses

**Fig 3:**
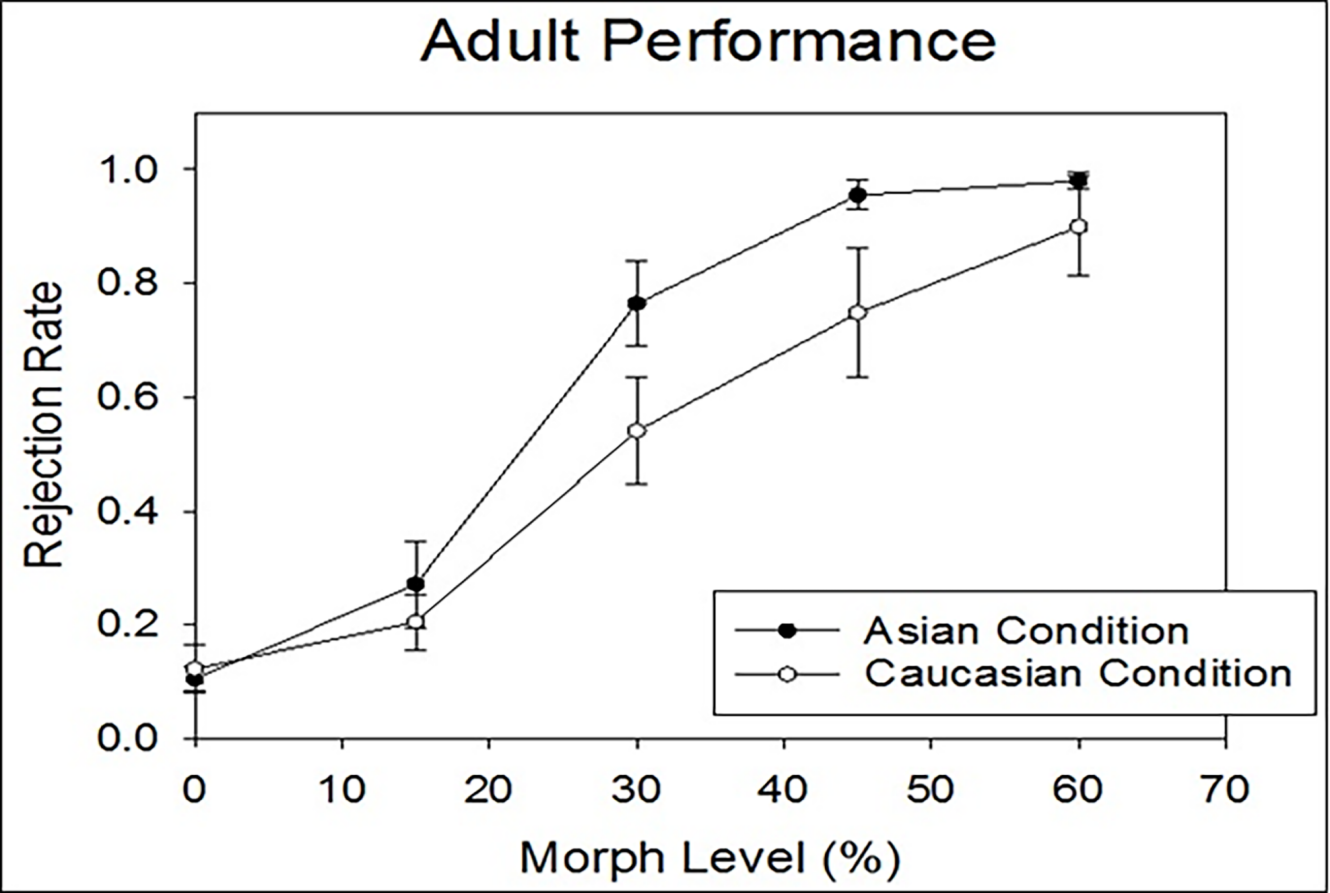
Adults’ mean rejection rates. The adults’ mean rejection rate (Y-axis) as a function of the morph level (X-axis) for the Asian-parent (black circles) and the Caucasian-parent condition (white circles). The rejection rate is the probability of judging the comparison face as “different” from the target face (i.e., the same as the *P*(diff) in Table 1). The morph level denotes the physical difference (in linear unit) between the target parent face and the comparison face.

**Fig 4:**
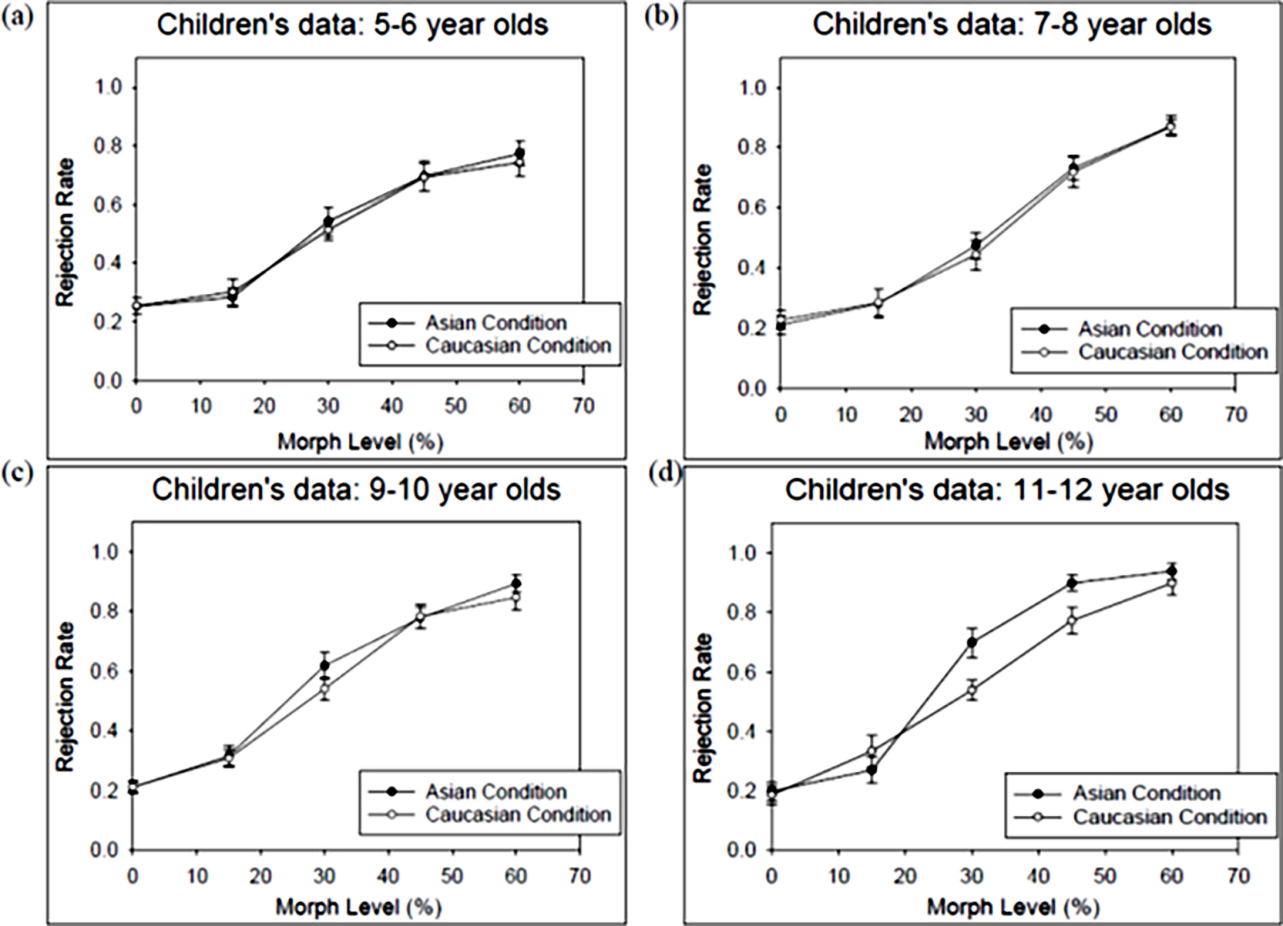
Children’s group mean rejection rates. The children’s group mean rejection rates (Y-axis) as a function of the morph level (X-axis) for the Asian-parent (black circles) and the Caucasian-parent condition (white circles). The rejection rate is the probability of judging the comparison face as “different” from the target face (i.e., the same as the *P*(diff) in Table 1). The morph level denotes the physical difference (in linear unit) between the target parent face and the comparison face. (a) 5-6-year-olds, (b) 7-8-year-olds, (c) 9-10-year-olds, (d) 11-12-year-olds.

To fully capture the characteristics and developmental changes in perceptual sensitivity for the Asian-parent and the Caucasian-parent conditions, we employed three data analysis methods applying to all age groups. First, as half of the trials were physically the same (i.e., 0% morph level) and half were physically different (all other morph levels), we adopted signal detection theory to code the participant’s four response categories (*hit*, *false alarm*, *miss*, and *correct rejections*), and compute d’ as an index of sensitivity based on the *hit rates* and *false alarms*. For a given age group, a higher mean d’ in the Asian-parent condition than that in the Caucasian-parent condition suggests an own-race encoding advantage. Moreover, an interaction between age and race would signal a developmental change in the own-race advantage. This set of data are shown on the right side of Table 1. Second, as there were four morph levels in the physically “different” trials (i.e., 15%, 30%, 45%, and 60%), we treated the morph level as a categorical variable and conducted an omnibus three-way ANOVA exploring the main effects of age group, race, and morph level as well as the interactions among them. Likewise, a significant interaction between age group and race would indicate a developmental change of the own-race encoding advantage. Please note that the second set of results would be highly similar to the first set of data analysis due to the overlap in the calculation of hit rates and d’s. This part of data is presented graphically in Figs 3 and 4, and numerically in the left five columns of Table 1.

**Table 1:** The group mean probability of judging the comparison faces as “different” (*P*(diff)) at the 5 morph levels and the computation using the signal detection theory (showing correct rejections, false alarms, misses, hits, and d primes) for the Asian-parent and Caucasian-parent conditions in children and adults. The numbers in the parenthesis denote the standard errors (SE) of the group means.

Age group	Asian-parent condition									
	*P*(diff) 0%	*P*(diff) 15%	*P*(diff) 30%	*P*(diff) 45%	*P*(diff) 60%	Correct Reject	False Alarm	Miss	Hit	*d'*
5–6 years	.25 (.03)	.28 (.03)	.54 (.05)	.70 (.05)	.77 (.04)	.75 (.03)	.25 (.03)	.43 (.04)	.57 (.04)	0.975 (.140)
7–8 years	.21 (.03)	.28 (.04)	.49 (.04)	.73 (.03)	.88 (.03)	.79 (.03)	.21 (.03)	.40 (.03)	.60 (.03)	1.229 (.126)
9–10 years	.21 (.02)	.32 (.03)	.62 (.04)	.78 (.04)	.89 (.02)	.79 (.02)	.21 (.02)	.35 (.02)	.65 (.02)	1.299 (.106)
11–12 years	.18 (.03)	.25 (.04)	.71 (.05)	.91 (.02)	.96 (.02)	.80 (.03)	.20 (.03)	.30 (.03)	.70 (.03)	1.591 (.124)
Adults	.10 (.02)	.26 (.04)	.75 (.04)	.92 (.01)	.98 (.01)	.90 (.03)	.10 (.03)	.26 (.02)	.74 (.02)	2.146 (.106)
Age group	Caucasian-parent condition									
	*P*(diff) 0%	*P*(diff) 15%	*P*(diff) 30%	*P*(diff) 45%	*P*(diff) 60%	Correct Reject	False Alarm	Miss	Hit	*d'*
5–6 years	.25 (.02)	.30 (.04)	.51 (.03)	.69 (.04)	.74 (.04)	.75 (.03)	.25 (.03)	.44 (.03)	.56 (.03)	0.928 (.134)
7–8 years	.23 (.03)	.30 (.04)	.46 (.05)	.73 (.05)	.87 (.02)	.77 (.04)	.23 (.04)	.41 (.03)	.59 (.03)	1.138 (.117)
9–10 years	.21 (.02)	.31 (.03)	.54 (.04)	.78 (.04)	.85 (.04)	.79 (.02)	.21 (.02)	.38 (.03)	.62 (.03)	1.186 (.113)
11–12 years	.19 (.03)	.33 (.05)	.54 (.04)	.77 (.04)	.90 (.04)	.81 (.03)	.19 (.03)	.36 (.03)	.64 (.03)	1.323 (.096)
Adults	.12 (.02)	.21 (.03)	.52 (.05)	.73 (.05)	.88 (.04)	.88 (.02)	.12 (.02)	.41 (.04)	.59 (.04)	1.552 (.124)

*Note*. *d’* was computed as Z(Hit)-Z(False Alarm). A standard correction is applied to the trials with hit rate of 1 and false alarm of 0 using 1-1/(2N) instead of 1.0 and 1/(2N) instead of 0. N = 36, which is the number of the physically same/different trials.

Last but not least, the morph level of the face stimuli can also be regarded as a continuous variable, and the performance dimension (i.e., the rejection rate) increases monotonically with the stimulus intensity (i.e., the morph level). Thus, we further fitted the group mean psychometric functions (X: the morph level, Y: the rejection rates) with a *cumulative normal distribution* model for the Asian- and the Caucasian-parent conditions separately. The curve fitting procedures allow us to estimate the group *discrimination threshold* (*μ*) (i.e., the minimum change in physical intensity required to produce a detectable change) and the group *slope* parameter (σ) (i.e., an index of response uncertainty. A sharp function means less internal noise or uncertainty) of the psychometric functions in children and adults. Therefore, for a given age group, the presence of a smaller discrimination threshold (or a sharper slope) in the Asian-parent condition than that in the Caucasian-parent condition would indicate the presence of an own-race advantage. The curve fitting data are summarized numerically in Table 2.

**Table 2:** The estimated discrimination thresholds (*μ*) and slope parameters (σ) of the group psychometric functions for the Asian-parent and Caucasian-parent conditions. The numbers in the parenthesis are standard errors (*SE*) of the estimates by *bootstrap resampling* with an iteration size of 200. Eleven iterations were performed for the adults’ psychometric functions, and ten iterations were performed for the children’s functions).

Age group	Asian-parent condition		Caucasian-parent condition	
	DT (*μ*)	Slope (σ)	DT (*μ*)	Slope (σ)
5–6 years old	28.85 (2.56)	36.87 (5.04)	29.77 (2.39)	39.74 (4.98)
7–8 years old	29.24 (1.82)	28.86 (3.05)	29.87 (2.38)	30.17 (4.09)
9–10 years old	24.29 (1.51)	26.99 (2.46)	26.52 (1.70)	28.95 (2.85)
11–12 years old	22.23 (2.37)	19.37 (3.50)	26.03 (0.72)	27.06 (1.16)
Young adults	21.58 (1.11)	13.00 (1.56)	29.72 (1.40)	22.33 (2.09)

*Note*. DT: Discrimination threshold, in percent morph level (%).

### Analyses of d’ for all age groups

In the current same/different face matching task, half of the trials were physically the same (i.e., 0% morph level) and half were physically different (i.e., 15%, 30%, 45%, and 60% morph levels). The participants would yield four possible response categories according to the Signal Detection Theory (SDT) [37, 38]. Conceptually, there were two types of accurate responses, the “*hits*” and the “*correct rejections*.” Judging the comparison face as “different” on the physically “different” trials was denoted as “*hit*” (i.e., successfully detected the presence of a new signal). Judging the comparison face to be the “same” on the physically same trials was denoted as “*correct rejections*.” Similarly, there were two types of incorrect responses, the “*miss*” and the “*false alarm*.” Judging the comparison face to be “the same as the target face” was denoted as “*miss*” for the physically “different” trials (i.e., fail to detect the presence of new signal). Lastly, judging the comparison face to be “different” on the physically “same” trials where the two face images were identical was denoted as “*false alarm*”.

The sensitivity index of d', defined as the Z(Hit)-Z(False Alarm), for the Asian-parent and Caucasian-parent conditions were computed separately. This sensitivity index tells us how well can adults and children distinguish between the briefly presented target faces and the following comparison faces. Table 1 illustrates the group average *hits*, *false alarms*, *misses*, *correction rejections*, and *d’s* for the Asian-parent and the Caucasian-parent conditions separately. A two-way mixed ANOVA was performed on the d’s with *Age group (*5–6 years old, 7–8 years old, 9–10 years old, 11–12 years old, and young adults) as the between-subject factor, and *Race* (Asian-parent vs. Caucasian-parent) as the within-subject factor. The *Age group* main effect was significant *F*(4, 121) = 10.496, *p* < .001, *η2 p* = .259; the group mean d’s increased with age which were 0.952 (*SE* = .103), 1.184 (*SE* = .101), 1.242 (*SE* = .097), 1.504 (*SE* = .114), and 1.834 (*SE* = .109) for the 5–6 yr olds, 7–8 yr olds, 9–10 yr olds, 11–12 yr olds, and young adults, respectively. The *Race* main effect was also significant *F*(1, 121) = 13.899, *p* < .001, *η2 p* = .104; the mean d’ was greater for the Asian-parent condition (*M* = 1.448, *SE* = .056) than that for the Caucasian-parent condition (*M* = 1.228, *SE* = .053).

Importantly, the Age group * Race interaction was significant *F*(4, 121) = 3.402, *p =* .011, *η2 p* = .102, indicating that the difference in the perceptual sensitivity for Asian versus Caucasian condition varies with age. To reveal whether the participants of certain age group exhibited an own-race encoding advantage—a higher mean d’ in the Asian-parent than that in the Caucasian-parent condition, we further analyzed the *Race* simple main effect for each of the five age groups. To adjust for *α* expansion, we set the error rate of each comparison at *α* level = .05/5 = .01. The adults showed a significantly higher d’ for the Asain-parent (*M* = 2.146, *SE* = .106) than that in the Caucasian-parent condition (*M* = 1.522, *SE* = .124), *t*(22) = 5.165, *p <* .001. In children, only the oldest 11-12-year-olds exhibited a marginally significant higher d’ for the Asain-parent (*M* = 1.591, *SE* = .124) than that in the Caucasian-parent condition (*M* = 1.363, *SE* = .096), *t*(20) = 1.497, *p =* .074. Children in the other age groups (5-6-, 7-8-, and 9-10-year-olds) did not show significant differences between the two race conditions.

To reveal whether a developmental change occurs—the performance improves with age for the Asian- and Caucasian-parent conditions, we also analyzed *Age group* simple main effects (as one-way ANOVA) for both race conditions. For the Asian-parent condition, the simple main effect of *Age group* was highly significant, *F*(4, 121) = 13.023, *p* < .001, indicating an overall improvement with age for the own-race condition. The Sheffe’s method was adopted (i.e., unequal N among age groups) to perform pairwise multiple comparisons and the *α* level was adjusted with the number of comparisons (*α* level = .05/10 = .005). The adults’ *d’* was significantly greater than the *d’*s of three groups of children; adults vs. 5–6 year-olds (*p* < .001), adults vs. 7–8 year-olds (*p <* .001), adults vs. 9–10 year-olds (*p* < .001), and was marginally greater than the oldest children, adults vs. 11–12 year-olds (*p* = .028). In addition, the difference between the youngest 5–6 year-olds and the oldest 11-12-year-olds was also marginally significant (*p* = .008). For the Caucasian-parent condition, the simple main effect of *Age group* was significant, *F*(4, 121) = 3.502, *p =* .01. However, the posthoc pairwise comparisons revealed that only the difference between the adults and the youngest 5–6 year-olds was marginally significant (*p =* .009).

In sum, the above analysis with the *d’* showed that an own-race advantage is present in the adults and the oldest children, but is still absent in children aged between 5 and 10. Moreover, for the Asian-parent condition, we observed an overall improvement in *d’* sensitivity from 5-year-olds to 11-year-olds and young adults. However, the increase in *d’* sensitivity was not as apparent in the Caucasian-parent condition.

### Analyses of rejection rates treating morph level as a categorical variable

As there were four morph levels in the physically “different” trials (i.e., 15%, 30%, 45%, and 60%) that were collapsed as “*hits*”in the SDT analyses, it is of great interest to know how well do children and adults perform at each morph level in the Asian- and Caucasian-parent conditions. Thus, we treated the morph level as a categorical variable. Our preliminary analyses revealed no significant difference between the performances of male and female participants for all age groups; we thus dropped the between-subject factor of *Gender* and conducted an omnibus three-way mixed ANOVA on the physically different trials only. The between-subject factor was *Age Group* (5–6 years old, 7–8 years old, 9–10 years old, 11–12 years old, and young adults), while the within-subject factors were *Race* (Asian-parent vs. Caucasian-parent) and *Morph Level* (15%, 30%, 45%, 60%). The dependent measure was the rejection rate (or P(diff)), defined as the probability that the participant judged the comparison face, of a given morph level, to be “different.” The group mean rejection rates at each morph level for both race conditions are shown in the left side of Table 1.

#### The main effects

The *Age group* main effect was significant *F*(4, 121) = 3.49, *p =* .01, *η2 p* = .104; the group mean rejection rates were .569 (*SE* = .024), .592 (*SE* = .023), .636 (*SE* = .022), .680 (*SE* = .027), and .661 (*SE* = .025) for the 5–6 yr olds, 7–8 yr olds, 9–10 yr olds, 11–12 yr olds, and young adults, respectively, showing a tendency of increasing rejection rate with age. The *Race* main effect was highly significant *F*(1, 121) = 12.910, *p* < .001, *η2 p* = .097; the mean rejection rate was greater for the Asian-parent condition (*M* = .652, *SE* = .012) than that for the Caucasian-parent condition (*M* = .602, *SE* = .014). This indicated that collapsed across the age groups and the morph levels, the probability of judging the comparison face to be different was higher in the Asian-parent condition. Lastly, the main effect of *Morph level* was significant, *F*(3, 363) = 468.76, *p*< .001, *η2 p* = .796, the rejection rate increased as the morph level of the comparison face increased; the mean rejection rates for the morph levels 15%, 30%, 45%, and 60% were .283 (*SE* = .015), .569 (*SE* = .016), .782 (*SE* = .016), and .877 (*SE* = .014) in order.

#### The interaction effects

One important goal of the present study was to reveal whether certain age groups exhibited an own-race advantage. Therefore, a significant interaction between *Age Group* and *Race* would suggest a developmental change in the own-race advantage. Indeed, the Age group*Race interaction was significant *F*(4, 121) = 3.135, *p =* .017, *η2 p* = .095, meaning that the difference in the rejection rates for Asian vs. Caucasian condition varies with age. To reveal whether certain age group exhibited an own-race encoding advantage—a higher mean rejection rate for the Asian-parent than that for the Caucasian-parent condition, we further analyzed the *Race* simple main effect (as paired-t-tests) for each of the five age groups, similar to the analyses with d’s stated above (the error rate was adjusted to *α* level = .05/5 = .01 as well). The adults showed a significantly higher rejection rate for the Asain-parent (*M* = .736, *SE* = .028) than that for the Caucasian-parent condition (*M* = .587, *SE* = .032), *t*(22) = 4.569, *p <* .001. Fig 3 illustrates the adults’ rejection rates as a function of the morph level of the two race conditions. In children, only the oldest 11-12-year-olds (Fig 4C) exhibited a marginally significant higher mean rejection rate for the Asain-parent condition (*M* = .712, *SE* = .025) than that for the Caucasian-parent condition (*M* = .650, *SE* = .026), *t*(20) = 1.889, *p =* .074. Children in the other age groups (5-6-, 7-8-, and 9-10-year-olds) did not show significant differences between the two race conditions. Fig 4 illustrates the four groups of children’s rejection rates as a function of the morph level of the two race conditions.

Likewise, we also analyzed the *Age group* simple main effects (as one-way ANOVA) for both race conditions to reveal whether a developmental change occurs. For the Asian-parent condition, the simple main effect of *Age group* was highly significant, *F*(4, 121) = 7.224, *p* < .001. Again, the Sheffe’s method was adopted to perform pairwise multiple comparisons, and the *α* level was adjusted to *α* level = .05/10 = .005. The youngest 5–6 year-olds’ mean rejection rate was marginally smaller than that of 9–10 year-olds (*p* = .049) and was significantly smaller than those of 11–12 year-olds (*p* = .002) and adults (*p <* .001). The 7–8 year-olds’ mean rejection rate was (marginally) smaller than 11–12 year-olds (*p* = .068), and significantly smaller than that of the adults (*p =* .008). The mean rejection rates of 9–10 year-olds, 11-12-year-olds, and the adults were not different from one another. Importantly, for the Caucasian-parent condition, the simple main effect of *Age group* was not significant, *F*(4, 121) = 0.878, *p =* .479.

The *Age group* *Morph level interaction was significant, *F*(12, 363) = 3.278, *p <* .001, *η2 p* = .099. As the morph level increased, the rejection rate increased more rapidly in adults and older children than in younger children. The group mean rejection rates for the morph levels 15%, 30%, 45%, and 60% were .293 (*SE* = .032), .529 (*SE* = .035), .696 (*SE* = .034), and .759 (*SE* = .030) for the 5–6 years olds; .289 (*SE* = .032), .473 (*SE* = .034), .732 (*SE* = .033), and .875 (*SE* = .030) or the 7–8 years olds; .312 (*SE* = .031), .579 (*SE* = .033), .781 (*SE* = .032), and .871 (*SE* = .029) in 9–10 years olds; .289 (*SE* = .037), .628 (*SE =* .040), .854 (*SE* = .039), and .948 (*SE* = .035) for the 11–12 years olds; and .233 (*SE* = .034), .634 (*SE =* .037), .846 (*SE* = .036), and .933 (*SE* = .032) for the adults, respectively. Lastly, the Race *Morph level interaction was also significant, *F*(3,363) = 7.017, p< .001, *η2 p* = .055. As the morph level increased, the rejection rates increased more rapidly in the Asian-parent condition than that in the Caucasian-parent condition. The group mean rejection rates across age groups at the morph levels 15%, 30%, 45%, and 60% were .279 (*SE* = .018), .622 (*SE* = .021), .816 (*SE* = .017), .897 (*SE* = .015) in the Asian-parent condition, and .287 (*SE* = .019), .516 (*SE* = .019), .747 (*SE* = .021), .857 (*SE* = .017) in the Caucasian-parent condition. The three-way Age group *Race*Morph level interaction was not significant.

In sum, similar to the findings with *d’* stated earlier, the analyses of Age group*Race interaction revealed the presence of an own-race advantage in the adults and the oldest children, but not in the younger children under age 10. For the Asian-parent condition, we observed an overall increase of the mean rejection rate from age 5 to age 10, and the oldest children’s mean rejection rate became adult-like. On the contrary, there was no significant developmental change in the mean rejection rate for the Caucasian-parent condition. Moreover, the other two interaction effects revealed that as the morph level increased, the rejection rate increased more rapidly in the Asian-parent than that in the Caucasian-parent condition, and more rapidly for adults and older children than for younger children.

### Group mean psychometric function curve fitting

In the last part of data analyses, we treated the *Morph Level* of the face stimuli as a continuous variable. The performance dimension was expressed as the probability of judging the face to be “different,” while the measurements were based on the number of discrete trials at the five different stimulus intensity, 0%, 15%, 30%, 45%, and 60%. We used Sigmaplot 13.0 to conduct global curve-fitting of the group-averaged psychometric functions for each age group. Data were fit by a *normal cumulative distribution function* (Normal CDF) for the Asian-parent and Caucasian-parent condition separately, with continuous points predicted by the formula below, to estimate 1) the *discrimination threshold* (*μ*), defined as the corresponding morph level yielding a rejection rate of 0.5, and 2) the *slope* parameter (σ).

$$y=\frac{1}{\sqrt{2\pi }\sigma }\int_{-\infty }^{x}e^{-\frac{{\left(x-\mu \right)}^{2}}{2\sigma^{2}}dx}$$

#### Comparisons between the two race conditions

Table 2 summarizes the estimated discrimination thresholds (*μ*) and the slope parameters (σ) for each age group. The numbers in the parenthesis are standard errors (*SE*) of the estimates by *bootstrap resampling* with an iteration size of 200 [39]. Eleven iterations were performed for the adults’ psychometric functions, and ten iterations were performed for the children’s functions. Based on the previous analyses, we learned that children below age 10 did not yet exhibit an own-race advantage. Thus, in the subsequent analyses, we primarily focused on the adults and the oldest 11-12-year-old children.

As shown in Table 2, the adults’ discrimination threshold (*μ*) was 21.58% in the Asian-parent condition and 29.72% in the Caucasian-parent condition. To reveal whether the difference was statistically significant, we used 95% confidence interval estimation (*μ*_*Asian*_ ± 1.96*SE_*Asian*_) basing on the threshold of the Asian condition (*μ*_*Asian*_) and asked whether the discrimination threshold of the Caucasian condition (*μ*_*caucasian*_) fell out of the confidence interval [39]. The lower limit of *μ*_*Asian*_ was 19.40%, the upper limit was 23.76%, and the threshold of the Caucasian condition *μ*_*caucasian*_ was greater than the upper limit (29.72%>23.76%). Hence, the difference was significant. Likewise, for the slope parameter (σ)—a measure that was in inverse proportion with the steepness of the function, we used 95% confidence interval estimation basing on the Asian condition (σ_*Asian*_ = 13.00 ± 1.96*SE_*Asian*_) to see whether the two slopes were different. Indeed, the slope parameter in the Caucasian condition (σ_*Caucasian*_ = 22.33) was far above the upper limit of the confidence interval (Lower limit: 9.94, Upper limit: 16.06). In sum, the above analyses showed that, Taiwanese adults not only needed a smaller physical difference (in the unit of morph level) to produce a detectable change but their group psychometric function also exhibited a steeper slope (i.e., a reduction in the internal noise or response uncertainty) for the own-race condition.

For the oldest 11-12-year-old group, the discrimination thresholds (*μ*) for the Asian- and the Caucasian-parent conditions were 22.63% and 26.03%. To reveal whether the 4% difference in morph level was statistically significant, we used 95% confidence interval estimation basing on the threshold of the Asian condition (*μ*_*Asian*_ ± 1.96*SE_*Asian*_). The lower limit was 17.58%, the upper limit was 26.88%, and the threshold of the Caucasian condition *μ*_*caucasian*_ was just below the upper limit (i.e., close to a “marginal” significance). Nevertheless, the slope parameters (σ) of the Asian condition (σ_*Asian*_ = 19.37) is approaching the adult’s level, whereas the slope parameter of the Caucasian condition remained close to other children’s (σ_*Caucasian*_ = 27.06). The 95% confidence interval estimation revealed that the σ_*Caucasian*_ = 27.06 was greater than the upper limit of the confidence interval (Lower limit: 12.51, Upper limit: 26.23), suggesting a steeper slope of the psychometric function for the own-race condition.

For children under age 10, the 5–6 years old exhibited a similar discrimination threshold (*μ*) for the Asian- and the Caucasian-parent conditions, 28.85% and 29.77%. The slope parameters (σ) of both conditions were similar and relatively shallow, 36.87 and 39.74, respectively. The 7–8 years olds’ discrimination thresholds (*μ*) for the Asian- and the Caucasian-parent conditions were 29.24% and 29.87%, and their slope parameters (σ) were 28.86 and 30.17, respectively. The 9–10 years old children’s discrimination thresholds (*μ*) for the Asian- and the Caucasian-parent conditions were 24.29% and 26.52%, and the slope parameters (σ) were 26.99 and 28.95, respectively.

#### Comparisons between the children and the adults

**Fig 5:**
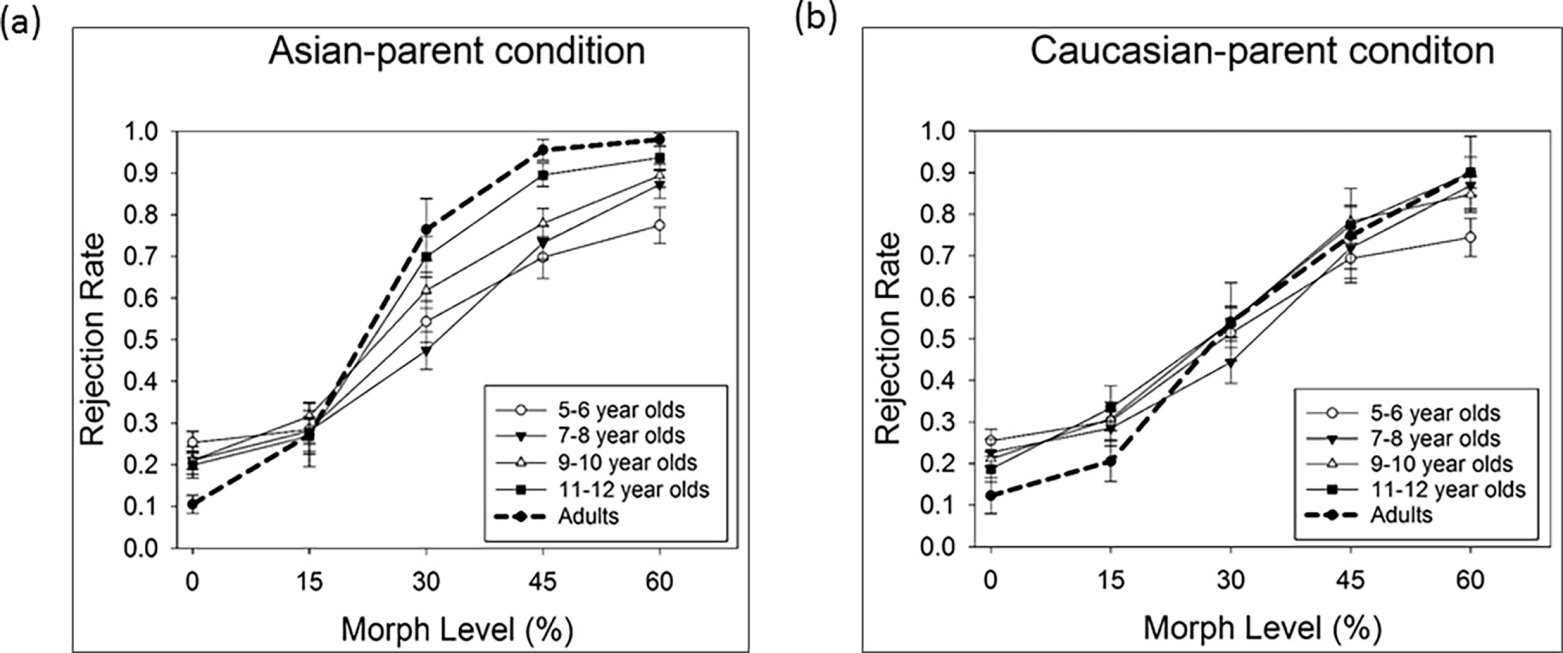
Comparing mean rejection rates among age groups. (a) The group mean rejection rate (Y-axis) as a function of the morph level (X-axis) in the Asian-parent condition, (b) and the Caucasian-parent condition. In each panel, the separate lines represent the performances of the different age groups with the adult’s data showing in bold dashed lines.

In this section, we explored whether a developmental change occurs in two possible forms: (1) if the discrimination threshold decreases with age, and (2) if the slope of the group psychometric function becomes steeper with age. To better present the developmental changes graphically, we put together the adults’ and children’s psychometric functions in one figure. Fig 5 illustrates the group psychometric functions for the Asian-parent condition (Fig 5A) and the Caucasian-parent condition (Fig 5B). For the Asian-parent condition, children from age 5 to 12 showed a gradual and continuous improvement in the perceptual discriminability for the Asian-parent condition. Such improvement included a left-ward shift of the psychometric function (lowering of the discrimination threshold in morph level unit) as well as sharpening of the slope (which is particularly evident for the oldest 11-12-year-old children).

The above descriptions were further endorsed by the 95% confidence interval estimation based on the adult’s threshold of the Asian condition. We asked whether the Children’s thresholds of the Asian condition fell out of the adult’s confidence interval, and found that all but the 11-12-year-old children’s discrimination thresholds were greater than the adult’s upper limit (>23.76%). Moreover, the 95% confidence interval estimation of the adult’s slope parameter showed that all children’s slope parameters were greater than the adult’s upper limit (>16.06). Furthermore, to reveal whether 11-12-year-old’s discrimination threshold was significantly smaller than those of other children, we used 95% confidence interval estimation and found that the thresholds of 5–6 year-olds, 7–8 year-olds, but not 9-10-year-olds were greater than the upper limit (> 26.88%). Similarly for the 95% confidence interval estimation of the slope parameter, all other children’s slope parameters exceeded the upper limit (>26.23) of the 11-12-year-old’s slope paramter.

Fig 5B summarizes the group performances in the Caucasian-parent condition. As opposed to the gradual and continuous improvement we found in the Asian-parent condition, children from age 5 to 12 and even in adults did not seem to show much improvement in discrimination threshold in the Caucasian-parent condition. The psychometric functions of each age groups, including the adults, largely overlapped with one another; this is especially clear for the data at the 15%, 30%, and 45% morph levels (i.e., a lack of left-ward shift). The 95% confidence interval estimation based on the adult’s threshold of the Asian condition indicated that indeed the thresholds of the Asian condition in all children fell within the adult’s confidence interval (Lower limit: 26.98%, Upper limit: 32.46%). Nevertheless, we did observe some sharpening of the slope between age 5 and 12, and the adult’s psychometric function was the steepest among all; they exhibited the smallest false alarm rate at the 0% morph level (the lower asymptote) and the highest rejection rate at the 60% morph level (the higher asymptote). The 95% confidence interval estimation of the adult’s slope parameter (Lower limit: 18.23, Upper limit: 26.43) showed that all children’s slope parameters were greater than the adult’s upper limit (>26.43). Comparing between 11-12-year-olds and the younger children, the 95% confidence interval estimation showed that the slope parameters of 5–6 year-olds, 7–8 year-olds, but not 9-10-year-olds were greater than the upper limit of the 11-12-year-olds’ slope parameter (Lower limit: 24.79, Upper limit: 29.33).

In short, the curve fitting analyses revealed that, while children aged between 5 and 10 performed equally well in both race conditions, the oldest 11-12-year-old children and adults showed better performance in the Asian-parent condition instead, indicating the presence of an own-race advantage in the latter two groups. The improvement in the perceptual sensitivity for the Asian-like morphed faces manifested itself in both lowering the discrimination threshold as well as sharpening the slope of the psychometric function, with the adults exhibiting the smallest threshold and the sharpest slope. On the other hand, the improvement in the Caucasian-like morphed faces was only observed in the slope parameter; the leftward shift in threshold was absent. Nevertheless, among all age groups, the adults still exhibited relatively better performance with the sharpest slope.

## Discussions

Using a sequential morphing face matching task with well-controlled physical similarities between the target and the comparison faces, the present study examined the encoding advantage hypothesis in Taiwanese school-aged children and young adults. Combining the signal detection theory analysis and psychometric function curve fitting, we characterized the perceptual sensitivity for own- versus other-race faces for each age group and obtained several important findings. First, the adults showed a significantly greater *d’*, a smaller discrimination threshold, and a sharper slop in the Asian-parent condition than those in the Caucasian-parent condition, indicating the presence of an own-race encoding advantage. This finding well agrees with the studies by Walker & Tanaka’s and Chen et al. [7,12], and supports Goldinger et al.’s [13] eye movement data showing an own-race bias in the initial encoding stage. Moreover, as the morphed images were created out of the same two parent faces in both race conditions, the above result could not be attributed to any inherent unequal physical similarities between the Asian- and Caucasian-parent faces.

Secondly, children aged between 5 and 10 showed about equal sensitivity index (*d’*) and discrimination thresholds for the Asian-parent and the Caucasian-parent conditions, indicating an absence of the own-race advantage for this age range. This is also revealed in Fig 4A, 4B, and 4C that children in the age range did not differ in the rejection rates between the two race conditions. Likewise in Table 2, these children showed fairly comparable discrimination thresholds in both race conditions. Nevertheless, compared to the 5-6-year olds and 7-8-year olds, 9-10-year olds showed some drop in the discrimination threshold of the Asian-parent condition and the slopes of both race conditions became steeper. Although children aged 5 to 10 has not yet exhibited a significant own-race advantage, their face matching performances were far from random—their rejection rates systematically increased as the physical dissimilarity between the target and comparison faces increased.

Thirdly, the oldest 11-12-year-old children started to show a higher d’ in the Asian-parent than in the Caucasian-parent condition. They also exhibited a (marginally) smaller threshold and a significantly sharper slope for the Asian-parent condition than those in the Caucasian-parent condition, indicating the presence of the own-race advantage. Not only they exhibited a superior performance in the Asian-parent condition, but also their psychometric functions began to look adult-like. Nevertheless, the oldest children’s proficiency in discriminating Asian-like morph faces (in terms of slope) has not yet fully reach the adult’s level.

Last but not least, when comparing among all age groups, we found two distinctive developmental trends: In the Asian-parent condition, children from age 5 to 12 showed a gradual and continuous refinement in the perceptual discriminability, and the adults exhibited the best performance. In the Caucasian-parent condition, on the other hand, very limited progress was observed, and the discrimination threshold did not improve much when even reaching adulthood (i.e., an absence of a leftward shift, only a sharpening of the slope). Broadly speaking, these findings are consistent with the results of Chance et al. and Goodman et al., both used a standard recognition memory paradigm[32, 33]. Chance et al. adopted Japanese and White faces to study the development of face recognition memory in Caucasian Children of grades 1–2 (i.e., 7-8-year-olds), 5–6 (i.e., 11-12-year-olds), 7–8 (i.e., 13-14-year-olds) and college students[32]. They found a presence for ORE in 11–12 year-olds (5–6 grades), teenagers (7–8 grades), and college students with the latter exhibiting the largest magnitude of the other-race effect. Secondly, they found an overall increase in recognition accuracy (in d prime) for both White and Japanese faces across, but the children’s d’ for recognizing own-race faces increased more rapidly with age. Similarly, Goodman et al. used a standard memory recognition task of Asian, African, and Caucasian faces and found the presence of ORE in 8- to 10-year-olds, 12- to 14-year-olds, and adults, but not in the youngest group of 5- to 7-year-old children [33]. Moreover, the recognition memory performance generally increased as a function of age, and the slope for recognizing own-race faces was much sharper than that for the other-race faces. Taken the two studies and the present one together, we observed an important developmental trend during the school-age years: children’s perceptual sensitivity for own-race face recognition increased substantially with age, while their perceptual sensitivity for own-race face recognition seemed to improve at a much slower pace.

One main finding that the emergence of the ORE, manifested as an encoding advantage for the own-race parent condition with the oval-cropped morphing faces, did not occur before 10 or 11 years of age seem to diverge from the results reported by Pezdek et al., Sangrigoli & de Schonen, and Suhrke et al. [29, 16, 28], who showed the presence of ORE between 3 and 5 years. Strictly speaking, we remain agnostic, since we did not directly test children aged between 3 and 5. Nevertheless, the inconsistent findings on the onset of ORE in the previous developmental literature could reflect various procedural discrepancies among the studies (i.e., encoding duration, retention interval). Another critical factor may relate to whether the selected stimuli were whole faces showing external cues, or oval-cropped faces with hair, ears, and external contours eliminated or covered. It has been reported that young children aged between 3 and 7 relied more on external features for face recognition [40]. A recent study manipulating the visibility of the external facial cues brought an insight into such discrepancy. Suhrke et al. tested 4-year- old German and Cameroon children’s recognition for Caucasian and African faces and found a modulation effect with headwear [41]. The ORE was present for both groups of children when tested with whole faces showing hair and facial contours. However, when tested with faces partially covered by headwear (which eliminated the external cues), Cameroonian children still exhibited an ORE, but German children did not, who were accustomed to seeing faces without headwear in their daily encounter.

Although face perception starts from birth, lines of evidence across decades suggested that children’s proficiency in processing faces has not yet reached the adults’ level. Carey and Diamond showed that accuracy in a match-to-sample task improves by about 20% from 6 to 10 years of age [42]. Germine et al. reported that from age 10 to 30 years, face recognition performance further improves by about 18% [43]. A recent study by Goffaux et al. explored the development of face processing across the lifespan [44]. They found that the size of the face inversion effect (FIE) linearly increased until young adulthood in the horizontal orientation range of face information. Likewise in the present study, children from age 5 to 12 showed a gradual and continuous progress in perceptual discriminability (i.e., lowering in the threshold) and a reduction in response uncertainty (i.e., sharpening of the slope) in the Asian-parent condition, the adults’ psychometric function was still the sharpest among all. The performance gap between the oldest children and the adults supported the view that an adult-like expertise in processing facial identity may take the entire childhood or even adolescent years to fully develop [40,42,44–48]. What is also important is that children’s improvement in perceptual sensitivity, in terms of reducing the discrimination threshold and increasing the steepness of the slope, is more prominent in the Asian-parent condition (own-race)—For the Caucasian-parent condition (other-race), very limited progress was seen in children from age 5 to 12, and the discrimination threshold did not further improve when reaching adulthood. Given the morphing faces used in the Asian- and Caucasian-parent condition were created from the same source with the same amount of physical similarities, the selective improvement in the own-race condition lends strong support for the *perceptual expertise hypothesis* (for a review, see Meissner & Brigham [2]; Young et al.[49]) or the *perceptual learning view* [20,50] (also see Lee et al. [51] for a recent review). Although there are many models and mechanisms associated with the *perceptual expertise hypothesis*, the very basic view shared the idea that our expert-like face processing ability for own-race faces does not generalize equally to all race faces. In other words, limited racial contact (or segregation) results in people developing better expertise in distinguishing between faces of their own familiar race than those of other unfamilar races.

What might be the reason that the ORE did not emerge until 10 or 11 years of age? Our best guess is that it seems to coincide with the age of children reported to exhibit an adult-like ability in face processing. One characteristic of the adult-like expertise in face recognition is the priority of using internal facial cues as opposed to using external cues. For example, Campbell et al. asked 4- to 10-year-old children to make familiarity judgment of part-face and whole-face of their schoolmates and found that external features were more accurate than internal features for younger children, with a switch to an adult-like pattern at age 9 or 10 [40]. In the context of the present study, a child must rely on information strictly pertaining to the internal part of the face to successfully discriminate the oval-cropped morphing faces of subtle differences.

Another important feature of an adult-like face processing is the mastering of configural processing [52]. In the present study, to detect the differences between the target parent face and a comparison morph face, the participants would have to rely more on a mixture of fine-grained second-order configural information (i.e., the spacing between the eyes) [53] and featural cues (i.e., changes in the shape of the eyes). Here the information of the first-order configuration would be less critical, since the first-order relation (i.e., the eyes are located above the mouth) was kept constant in any given morphing face. Mondloch et al. reported a well-controlled study where a single face was altered to create sets of faces designed to measure featural, configural, and contour processing in 6- to- 10-year-old children and adults [47]. They found that, on the featural set, adults and children of all age performed equally well. However, on the spacing set,10-year olds outperformed the younger children but still made more errors than the adults. This suggested that the configural processing developed more slowly than featural processing and did not emerge until the age of 10. Although we do not mean to draw a direct connection between the emergence of the second order configural processing and the emergence of ORE in child development, we do observe a noteworthy coincidence that this particular age, 10 or 11, begins to show an adult-like expertise in face recognition. This coincidence may give us clues to explain why that the ORE did not emerge until 10 or 11 years of age in the present study.

In conclusion, the present study is not the first to adopt the morph face paradigm in studying the other-race effect, but perhaps is the first to examine school-age children’s perceptual sensitivity for own- and other-race morph faces by estimating the discrimination thresholds and slope parameters with psychophysical methods. Our results support the view that expertise in face processing takes the entire childhood to fully develop, and the emergence of the own-race encoding advantage coincides well with the onset of an adult-like expertise in processing face identity. Lastly, the other-race effect seen in adulthood may reflect a result of prolonged learning specific to own-race faces most commonly seen in one’s visual environment—a finding supporting the *perceptual expertise* or *perceptual learning* hypothesis of ORE. Important avenues for future research are to extend the age range to adolescence, to increase sample size and regard age as a continuous variable, to obtain convergent evidence of participants from a different race/ethnicity, and to explore how the visual own-race bias may link to the development of race-based social preference/prejudice of “in-group” vs. “out-group” distinction [54, 51].
